# Characterizing Charge Structure in Central Argentina Thunderstorms During RELAMPAGO Utilizing a New Charge Layer Polarity Identification Method

**DOI:** 10.1029/2021EA001803

**Published:** 2021-08-18

**Authors:** Bruno L. Medina, Lawrence D. Carey, Timothy J. Lang, Phillip M. Bitzer, Wiebke Deierling, Yanan Zhu

**Affiliations:** ^1^ Department of Atmospheric and Earth Science The University of Alabama in Huntsville Huntsville AL USA; ^2^ NASA Marshall Space Flight Center Huntsville AL USA; ^3^ University of Colorado Boulder Boulder CO USA; ^4^ Earth System Science Center The University of Alabama in Huntsville Huntsville AL USA

## Abstract

A new automated method to retrieve charge layer polarity from flashes, named Chargepol, is presented in this paper. Using data from the NASA Lightning Mapping Array (LMA) deployed during the Remote sensing of Electrification, Lightning, And Mesoscale/microscale Processes with Adaptive Ground Observations (RELAMPAGO) field campaign in Cordoba, Argentina, from November 2018 to April 2019, this method estimates the polarity of vertical charge distributions and their altitudes and thicknesses (or vertical depth) using the very‐high frequency (VHF) source emissions detected by LMAs. When this method is applied to LMA data for extended periods of time, it is capable of inferring a storm's bulk electrical charge structure throughout its life cycle. This method reliably predicted the polarity of charge within which lightning flashes propagated and was validated in comparison to methods that require manual assignment of polarities via visual inspection of VHF lightning sources. Examples of normal and anomalous charge structures retrieved using Chargepol for storms in Central Argentina during RELAMPAGO are presented for the first time. Application of Chargepol to five months of LMA data in Central Argentina and several locations in the United States allowed for the characterization of the charge structure in these regions and for a reliable comparison using the same methodology. About 13.3% of Cordoba thunderstorms were defined by an anomalous charge structure, slightly higher than in Oklahoma (12.5%) and West Texas (11.1%), higher than Alabama (7.3%), and considerably lower than in Colorado (82.6%). Some of the Cordoba anomalous thunderstorms presented enhanced low‐level positive charge, a feature rarely if ever observed in Colorado thunderstorms.

## Key Points

- A new automated method to estimate the polarity, altitude, and vertical depth of charge layers from flashes is presented
- Thunderstorm charge structures in Central Argentina are characterized from months of Lightning Mapping Array (LMA) data, with 13.3% anomalous charge structure
- Cordoba anomalously charged thunderstorms have a distinct charge altitude distribution when compared to those in Colorado

## 1 Introduction

Past studies have associated the severity of thunderstorms with patterns in charge distribution (Fuchs et al., 2018; Wiens et al., 2005). The dominant meteorological environment provides initial conditions that would influence the kinematics and microphysics within thunderstorms, which in turn affects its charge structure and dominant cloud‐to‐ground lightning (CG) polarity. Relatively few studies have documented the charge structure over continents other than North America (López et al., 2019; Pawar & Kamra, 2004; Pineda et al., 2016; Qie et al., 2005). Therefore, documenting charge structures of storms in regions such as Argentina, which has some of the highest flash rates in thunderstorms in the world (Zipser et al., 2006), is of interest for develop a more robust understanding of global storm charge structures.

Due to the nature of lightning processes and their characteristic emission in the very‐high frequency (VHF) spectrum, thunderstorm charge structures associated with flashes can be inferred from Lightning Mapping Array (LMA) observations (Lang & Rutledge, 2011; Rust et al., 2005; Wiens et al., 2005). Based on knowledge of radiation propagation by lightning, VHF‐based sensors primarily detect radiation from negative breakdown of lightning that propagates through regions of positive charge (Mazur & Ruhnke, 1993; Rison et al., 1999). Then, mapping of VHF sources is used to manually determine the location of positive and negative charge layers (Bruning et al., 2007; Lang and Rutledge, 2008; Rust et al., 2005; Wiens et al., 2005). An intra‐cloud lightning (IC) flash initiates in a region with strong electric field, in between regions of charge with opposite polarities. Upon initiation, bi‐directional leaders are formed and move into opposite regions of charge: a positive leader moves to a region of net negative charge, and a negative leader moves to a region of positive charge in the cloud (Coleman et al., 2003; Kasemir, 1960). When a leader reaches a charge layer, it propagates horizontally through the charge layer away from the flash initiation location (Shao & Krehbiel, 1996). Flashes propagating through charge regions that constitute a vertical dipole with positive charge located above negative charge, are referred to as positive cloud flashes (+IC), while flashes that propagate through negative over positive dipoles are defined as negative cloud flashes (−IC, Bruning et al., 2014). K‐processes may also occur, transporting charge to the base of the initial channel (Shao & Krehbiel, 1996). Observations of the VHF source altitude distributions for long periods of time are generally used to infer the location of charge regions, as the altitude with most sources are often associated with positive charge layers (Fuchs & Rutledge, 2018; Fuchs et al., 2018; Lang & Rutledge, 2011; Lang et al., 2020). Tessendorf et al. (2007b) infer charge layer polarity automatically by using the first LMA source altitude for a flash, and the number of sources above and below that altitude. Stough and Carey (2020) utilized the DBSCAN (Density‐Based Spatial Clustering of Applications with Noise, Ester et al., 1996) algorithm to identify regions of dense sources and infer charge region polarity. Electric field soundings have been deployed to infer polarity of charge regions within and nearby thunderstorms (Marshall et al., 1995; Rust & MacGorman, 2002; Stolzenburg et al., 1998), and have been compared to LMA‐inferred charge regions (Rust et al., 2005). In order to interpret an electric field data set with altitude, the Gauss' Law approximation is assumed, where the charge density is proportional to the electric field variation with height (Stolzenburg et al., 1998).

The charge regions between which lightning initiates, and propagate, are formed early into a storm's lifetime. These charge regions are a result of a non‐inductive charging (NIC) mechanism, which does not require a pre‐existing electric field to polarize the cloud and precipitation size particles. In the NIC mechanism, the polarity that graupel particles acquire when colliding with ice crystals in the presence of supercooled liquid water (Saunders et al., 1991; Takahashi, 1978) depends on the temperature, and the effective liquid water content (EWC, the accreted fraction of the liquid water content). High (low) temperature and large (small) EWC are associated with graupel charging positively (negatively), and ice crystals charging negatively (positively) (Berdeklis & List, 2001; Pereyra et al., 2000; Saunders & Peck, 1998; Saunders et al., 1991, 2001; Takahashi, 1978). As the rimer particle (e.g., graupel) accretes supercooled liquid water, it is heated by latent heat, which sublimates the ice surface and reduces the diffusional growth (Williams et al., 1991). According to the relative diffusional growth theory (Baker et al., 1987; Emersic & Saunders, 2010), the ice particle growing faster by diffusion acquires positive charge. Particle differential fall speeds and updrafts lead to storm‐scale charge separation, with ice crystals being transported upward to cloud tops, and graupel residing in the mixed‐phase region in the mid‐levels forming the two largest charge regions during the developing‐to‐mature stage of thunderstorms (Williams, 1985).

Thunderstorms with upper‐level negative and mid‐level positive charge layers define an anomalous charge structure, as observed in thunderstorms during the STEPS field campaign conducted in Kansas, Colorado, and Nebraska (MacGorman et al., 2005; Rust & MacGorman, 2002; Rust et al., 2005; Tessendorf et al., 2007a, 2007b; Weiss et al., 2008; Wiens et al., 2005). They have also been observed in thunderstorms in Oklahoma by Emersic et al. (2011) and Marshall et al. (1995), during the TELEX field campaign (MacGorman et al., 2008), in Texas (Chmielewski et al., 2018), Alabama (Stough & Carey, 2020), and Spain (Pineda et al., 2016). Storms with a normal charge structure would have a dominant net negative charge in the mixed‐phase layer, and net positive above, as demonstrated in early foundational studies reviewed by Williams (1985), in the in‐situ aircraft studies by Dye et al. (1986, 1988, 1989), during TELEX (Bruning et al., 2007) and STEPS (Weiss et al., 2008) field campaigns, among others. A low‐level charge layer with opposite polarity to the nearest charge region is occasionally present (López et al., 2019; Pawar & Kamra, 2004; Williams, 1989) and, if positive and abnormally large, may also be termed anomalous (Bruning et al., 2014; Fuchs et al., 2015; Qie et al., 2005). Some events can have multiple charge regions, such as mesoscale convective systems (MCSs) (Lang & Rutledge, 2008; Lund et al., 2009; Stolzenburg et al., 1998), multicell storms (Bruning et al., 2007), and supercells (Bruning et al., 2010; Calhoun et al., 2013; Wiens et al., 2005).

Fuchs and Rutledge (2018) analyzed a large lightning flash data set for isolated cells in four different regions in the United States, and found that Colorado storms have a prevalence of anomalous charge structures compared to other regions. Colorado's highest flash rate mode was observed at lower levels (warmer temperatures and higher radar reflectivity values) than in other regions. In addition, they suggested that Colorado is followed by Oklahoma in terms of anomalous storm frequency, followed by Alabama and Washington D.C. with rare anomalous observations. A large occurrence of positive cloud‐to‐ground lightning (+CG) is often associated with anomalous charge structure storms, as a main net positive charge region is at the middle or low levels of a storm instead of near its top, facilitating the propagation of positive leaders toward the ground, especially if a small opposite (negative) charge region is present at lower levels. Orville and Huffines (2001) found that the percentage of +CGs in the United States varies from 2% in Florida to 10%–20% in a region extending from the High Plains of Eastern Colorado to the Upper Midwest. In the central and north Great Plains, a high percentage (>50%) of severe storm reports were found to be associated with predominantly +CG lightning (>50% of CGs being positive), when compared to southern Great Plains and eastern United States (Carey et al., 2003).

This study aims to characterize the charge structure in the Central Argentina region for the first time, utilizing a large data set, as it is a key science goal of the RELAMPAGO (Remote sensing of Electrification, Lightning, And Mesoscale/microscale Processes with Adaptive Ground Observations) field campaign (Nesbitt et al., 2021). This novel research is achieved by first developing and testing a new automated method to retrieve thunderstorm charge layer polarity using LMA source and flash data, which is described in this paper. Southeast South America has among the most severe thunderstorms in the world in terms of high flash rate (Zipser et al., 2006), hail size (Cecil & Blankenship, 2012), heavy precipitation, and flash floods (Rasmussen et al., 2014). Lightning characteristics have only been documented using LMA data recently in this region (Lang et al., 2020), and the distribution of charge within Argentina thunderstorms is explored for the first time in great detail in this study. The general charge structure is estimated for a large data set with a new algorithm, allowing for the inference of the likelihood of normal and anomalous charge structure. Similar to Tessendorf et al. (2007b) and Stough and Carey (2020), this method automatically infers charge polarity from flashes, more closely resembling Tessendorf et al. (2007b) method but with improved procedures, better emulating the steps that a human expert would perform when assigning polarity to LMA sources for a flash by detecting the negative leader in a bi‐directional model and assigning polarity to sources of a flash (e.g., Rust et al., 2005). In this study, if a given lightning flash passes a series of conditions, an algorithm analyzes its source location and time in order to produce a prediction of charge layer polarity for that flash. This method has the capability to be quickly applied to a large number of lightning flashes in a large LMA data set (e.g., 0–10 min to process 24 h of LMA flash level data within 100 km of the network center), which allows for the inference of the general charge structure and its evolution in time for a thunderstorm or for a large area of interest, as demonstrated by examples in this paper. The new algorithm infers three‐dimensional charge distribution on the flash level but its output is simplified to vertical charge layer profiles for the science applications highlighted in this study. Hence, output of this method is similar to manual assignment of polarity, providing positive and negative layer altitude and vertical depth, but it is much less labor intensive. This algorithm provides a detailed inference of the charge layer distribution in the vertical, including altitude and vertical depth of negative charge layers, which is often not possible to be analyzed from the VHF source distribution analysis, a method in which positive charge altitude is inferred from its peak distribution. Lastly, this paper will present a detailed application of the new charge layer polarity algorithm by characterizing the charge structure of Central Argentinian thunderstorms by processing a large multi‐month sample of LMA observations for the first time. The algorithm performance is then further demonstrated through its application to multi‐month LMA datasets from several locations in the United States in which charge structure has already been documented using the LMA‐based charge layer retrieval techniques discussed above. The additional application herein allows the charge structure of Central Argentinian thunderstorms to be compared for the first time to several well‐studied locations in the United States such as Colorado, Oklahoma, West Texas and Alabama using the same algorithm. Consistency with prior studies of charge structure in well observed regions of the United States ensures that this method is applicable for future work.

## 2 Lightning Networks Deployed During RELAMPAGO and DC3

The LMA is a GPS‐based network (Goodman et al., 2005; Koshak et al., 2004; Krehbiel et al., 2000; Rison et al., 1999) that operates in the VHF electromagnetic spectrum (Krehbiel et al., 2000), in which radiation events detected are often associated with lightning breakdown processes (Rison et al., 1999). LMAs locate and report the time of VHF sources emitted during lightning breakdown processes using a time‐of‐arrival technique (Koshak & Solakiewicz, 1996; Koshak et al., 2004; Lhermitte & Krehbiel, 1979; Thomas et al., 2004), where a χ^2^ goodness‐of‐fit function with a threshold of lower than five is utilized to minimize location errors, and minimum of six operating network sensors are required to ensure location accuracy (Chmielewski & Bruning, 2016). The lmatools Python package (Bruning, 2015) was used to process LMA source data into lightning flash datasets. This package is based on the DBSCAN (Ester et al., 1996) algorithm, a machine learning algorithm used to cluster VHF sources to reconstruct the shapes (structure) of entire lightning flashes. DBSCAN randomly searches for clusters of VHF sources in space and time, and groups each cluster individually. These groups are used to define individual flashes using the following criteria: source‐to‐source minimum distance and time thresholds of 3,000 meters and 150 ms, respectively, and a maximum flash duration of 3 s (Fuchs et al., 2016).

As part of the RELAMPAGO field campaign (Nesbitt et al., 2021), an LMA of 11 sensors was deployed by NASA Marshall Space Flight Center to the eastern side of the Sierra de Cordoba mountains in the province of Cordoba, Central Argentina, from mid‐November 2018 to mid‐April 2019 (Lang et al., 2020). RELAMPAGO LMA data was used in this study for development and validation of the charge layer inference method, and characterization of the charge structure climatology in the Cordoba warm season. In addition, LMA datasets from the DC3 (Deep Convective Clouds and Chemistry, Barth et al., 2015) field campaign are used to independently estimate the charge structure in a variety of climatological regimes of the United States to compare results of the presented algorithm with those of other studies which relied on manual charge inference (analysis), and with storms in the Cordoba region of Argentina examined during RELAMPAGO. During the DC3 field campaign, LMA networks were deployed simultaneously in Alabama, West Texas, Oklahoma, and Colorado in May and June 2012 (Barth et al., 2015; DiGangi et al., 2016; Mecikalski et al., 2015). For each data set, only flashes with centroid location within the 100 km range distance from the LMA network center are being considered in this study, as altitude errors are expected to be small (−3.9 m) and the flash detection efficiency to be about 95% (Chmielewski & Bruning, 2016; Koshak et al., 2004; Lang et al., 2020; Thomas et al., 2004) within this range. In addition, flashes with less than 20 detected sources were not considered in this study (see Section 3 for details).

## 3 Description of the Charge Layer Polarity Identification Method

**Figure 1:**
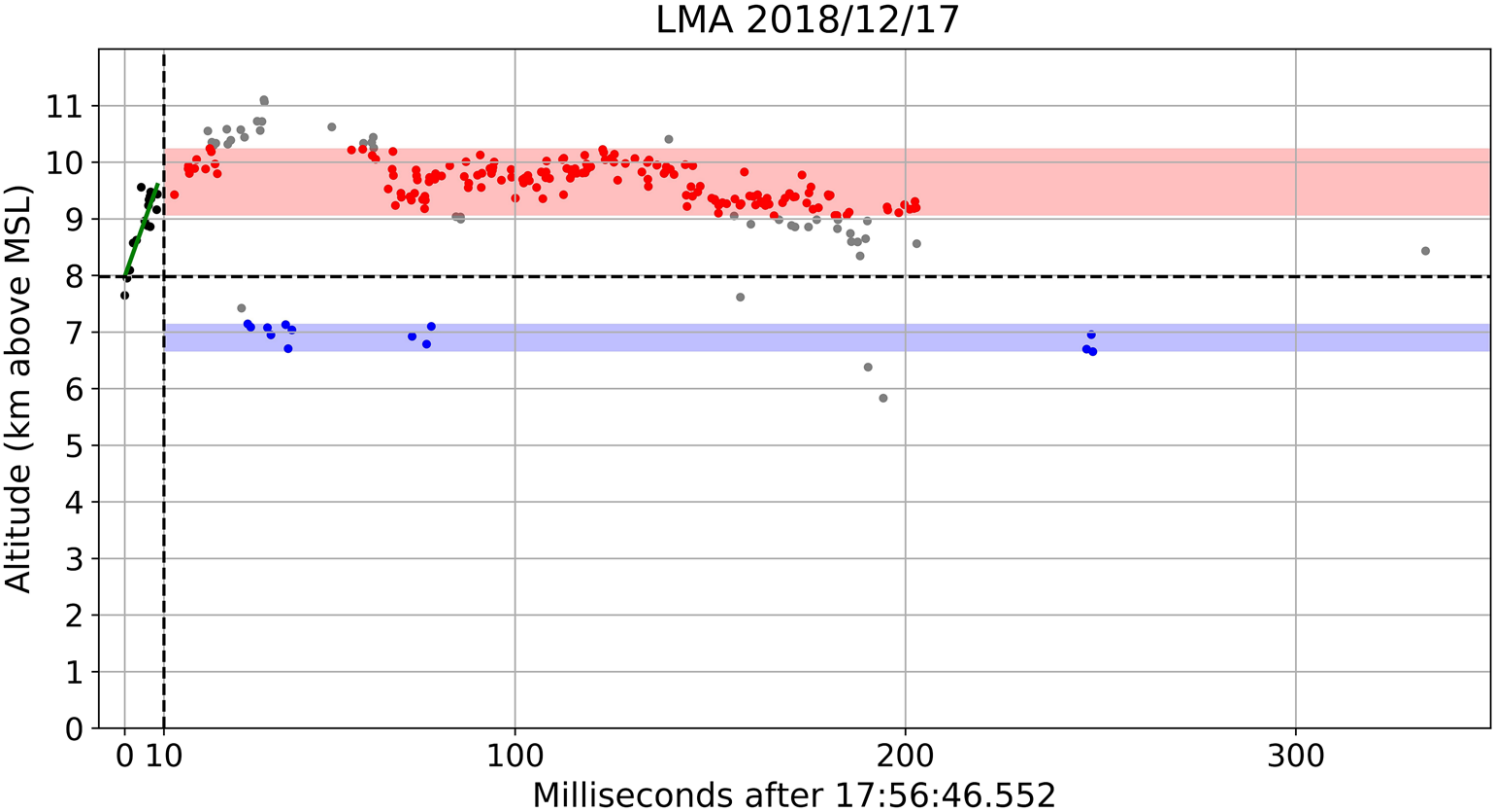
A time‐height plot for a positive intracloud flash. The vertical dashed line marks the 10 ms time limit that defines the Preliminary Breakdown (PB) sources (black dots). The green line is the linear regression fit on the PB sources. The horizontal dashed line is the CHT (Charge Height Threshold) that separates candidate sources for positive and negative charge layers. Red and blue dots (and shaded areas) define the positive and negative charge layers altitudes and width for this flash, found by applying the interval between the 10^th^ and 90^th^ percentile source altitudes for each polarity candidate sources. Gray dots are candidate sources outside the 10^th^–90^th^ percentile interval, which were not used to define charge layers.

The charge layer polarity identification method (hereafter Chargepol) consists of an automated algorithm that applies a series of procedures to each lightning flash retrieved by the lmatools, in order to infer charge layer polarity from a flash (link in the Acknowledgments). For reference, Figure 1 shows a flash example with the procedures illustrated. First, flashes with less than 20 sources are disregarded because those flashes would not allow a sufficient number of sources to characterize the initial negative leader breakdown, negative leader propagation through a positive charge region, and sources associated with a negative charge region. Then, all sources contained in the first 10 ms of a flash, referred to here as the Preliminary Breakdown sources (PB sources), are analyzed. A minimum of four PB sources is required, and the time interval between the first and last PB source has to be at least 2 ms, in order to better characterize the initial vertical motion of the negative leader. Typical duration periods are between 4 and 10 ms for PB (Zheng et al., 2019). We make the assumption that PB sources are associated with negative breakdown having a predominant vertical motion toward a region of positive charge (Shao & Krehbiel, 1996). Hence, linear regression is applied to the PB sources time‐height dimension. The linear regression slope is used as a proxy for the vertical speed of the leader, and has to be greater than a threshold of absolute value of 0.05 (0.5 km height variation in 10 ms), which is equivalent to a vertical speed of 5 × 10^4^ m s^−1^, or half the typical order of magnitude speed of a negative leader (Behnke et al., 2005; Shao & Krehbiel, 1996; van der Velde & Montanya, 2013). By applying that slope threshold, flashes with no clear initial vertical motion are discarded, further facilitating a plausible depiction of charge region polarity. In addition, the linear regression fit to the PB sources required a mean squared error (MSE) of less than 0.25 to prevent fitting a regression to noisy sources.

Only flashes that satisfy all the aforementioned conditions are used for charge layer depiction, which is typically about 16% of all flashes (more on this in Section 5). The fact that not all flashes are analyzed does not interfere with the objective of this study, because estimating charge polarity for some flashes is sufficient to determine the charge structure evolution over long periods of many hours, as demonstrated in the next section. Next, non‐PB sources (sources after the PB 10 ms threshold from flash initiation) are used to infer charge layer polarity, altitude and vertical depth. The PB linear regression intercept altitude is used as a threshold, referred to here as the Charge Height Threshold (CHT), in order to separate positive and negative charge layers candidate sources. For a positive PB linear regression slope (i.e., a flash with initial negative leader moving upward), all non‐PB sources above (below) the CHT are candidate sources to define a positive (negative) charge layer. A flash with initial downward motion (negative PB linear regression slope) would have all non‐PB sources below (above) the CHT as candidate sources for positive (negative) charge layer. Then, among the candidate sources for each layer polarity, the interval between the 10^th^ and the 90^th^ percentile source heights is used to define a charge layer, which provided a better distinction between positive and negative vertical histograms when compared to other intervals and also neglected sources with possibly doubtful polarity interpretation. For some flashes, it is possible that only one polarity layer is estimated, which leads to the total number of estimated positive layers from flashes for a large period of time being slightly larger than the number of estimated negative layers from flashes.

### 3.1 Validation Using Manual Analysis of LMA

**Figure 2:**
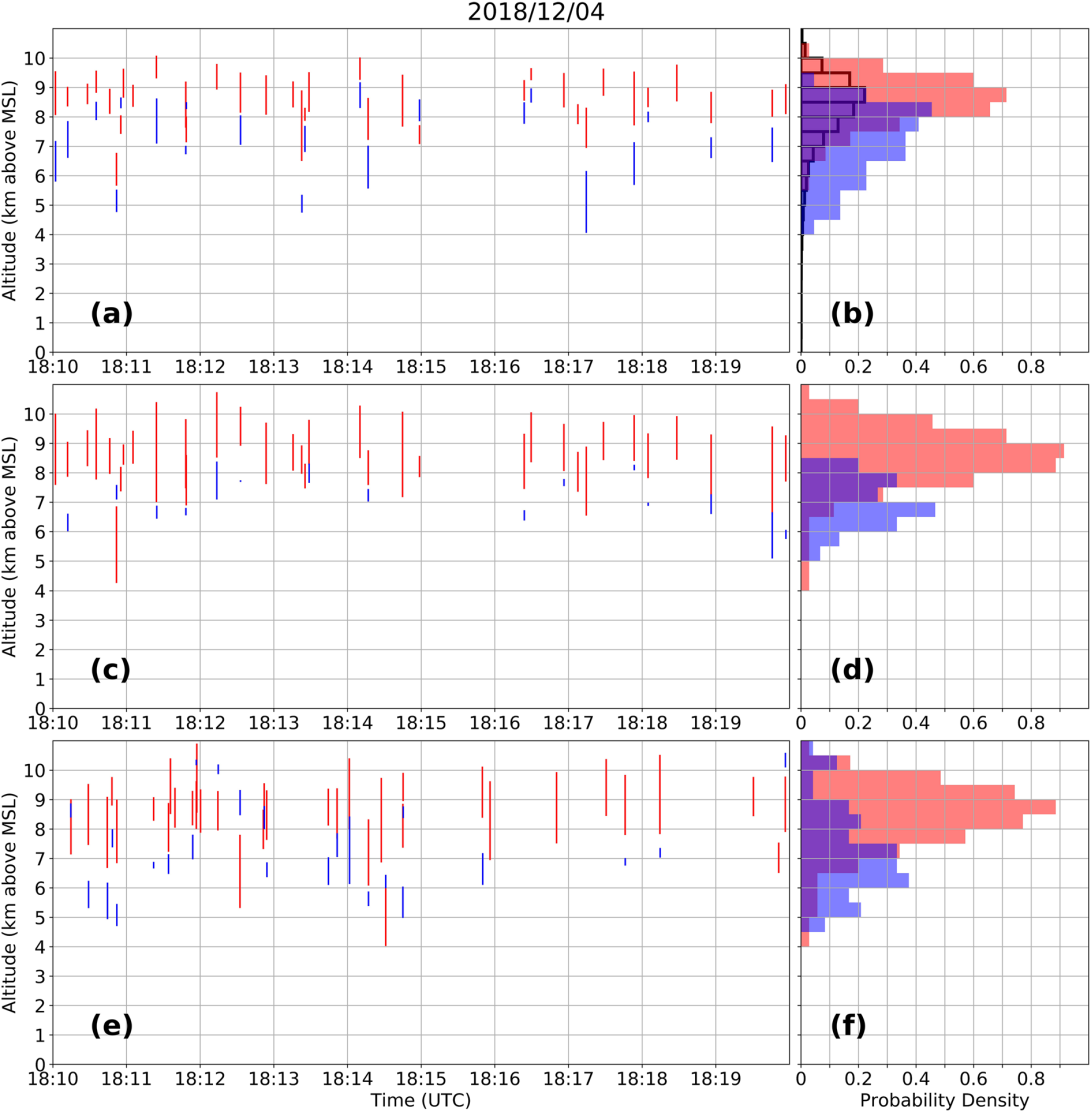
(a) Charge layers estimated from 35 flashes using the Chargepol automated method, (c) polarity assigned manually for the same 35 flashes considered by Chargepol, and (e) polarity assigned manually for 35 other flashes not considered by Chargepol during the same time period. Each red (blue) vertical line represents a positive (negative) charge layer estimated from a flash. (b), (d), and (f) shows histograms (0.5 km bin size) with the probability density of retrieved positive and negative charge layers with height for (a), (c), and (e), respectively (overlap of histograms in purple). Black histogram in (b) shows the source height distribution. The 10 min period chosen had a predominance of normal charge structure as clearly shown by the Chargepol algorithm, manual analysis, and source distribution.

In order to validate the automated Chargepol identification method, manual polarity inference (Rust et al., 2005; Wiens et al., 2005) was performed on some lightning flashes, and compared with the Chargepol algorithm output. First, a 10 min period (December 4 from 1810 to 1820 UTC) with a predominance of normal charge structure (i.e., normal dipole with positive charge over negative charge) was chosen from the RELAMPAGO LMA data set. Among the 168 flashes that occurred in this period with a normal charge structure, the algorithm estimated charge layers from 35 of them (21%) (Figure 2a). Figure 2b shows a histogram density with the altitude where each charge layer polarity was detected (a peak in the probability of positive sources being between 8.5 and 9 km height of 0.7 means that 70% of all positive charge occurred at that level). Source polarities were manually assigned for the same 35 flashes, shown in Figures 2c and 2d. The positive charge altitude was estimated to be between about 8 and 9.5 km from both Chargepol (Figure 2b) and the manual method (Figure 2d). Manual assignment of negative charge (Figure 2d) proved to be challenging, as it could not be estimated from all lightning flashes. Even so, it is notable that the negative charge layer is located at altitudes generally below the altitude of positive charge, with peak occurrence between 6.5 and 7 km height (Figure 2d). Additional validation was performed by assigning polarity for another 35 randomly chosen flashes among the 133 flashes during the same 10 min period that were not considered by Chargepol (Figure 2e). Most of these flashes did not have a clear vertical trend of the initial leader (not shown). However, as shown in Figure 2f, most positive charge layer detections from flashes were estimated to be between 8 and 9.5 km, consistent with the automated method (Figure 2b), while negative charge is located at lower altitudes. The analysis of an independent subset of flashes from Figures 2e and 2f demonstrates that Chargepol analysis on a fraction of total flashes is sufficient for the determination of thunderstorm charge structure.

**Figure 3:**
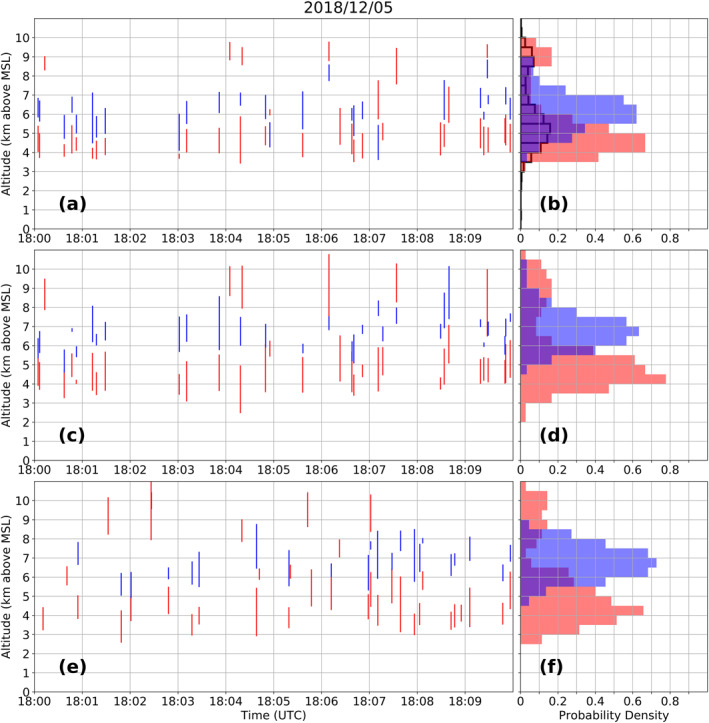
Same as in Figure 2, but for a 10 min period with predominance of anomalous charge structure.

This procedure was repeated for a 10 min period (December 5 from 1800 to 1810 UTC) with a predominance of anomalous charge structure (dipole with positive charge located below negative charge), shown in Figure 3. During this period, a high flash rate storm produced mostly negative ICs propagating through a lower positive charge. Another storm with low flash rate and upper positive charge layer was active at the same time. A total of 107 flashes occurred during this period, in which Chargepol estimated charge layers for 36 of them (Figures 3a and 3b). Manual depiction of charge polarity for these same 36 flashes (Figure 3c) show that the altitudes of positive and negative charge layers (Figure 3d) are in agreement with Chargepol, although manual inference of negative charge is at a slightly higher altitude. From Figure 3d, more than 50% of the negative charge layers occurred at altitudes from 6 to 7.5 km, while Chargepol estimated negative charge layers from 5.5 to 7 km height (Figure 3b). The small differences in charge layer altitudes between the manual and automated method demonstrate the small uncertainty of the method. Manual inference for a different set of 36 flashes during the same time period that was not considered by Chargepol is shown in Figures 3e and 3f, and it is consistent with other flashes (Figures 3a and 3c) in locating lower positive charge and mid‐level negative charge. The altitude distance between positive and negative charge layers centers (from histogram plots) for all methods is about 2 km. The few upper positive charge layers located above 8.5 km by all methods are from the normal charge structure storm.

The manual inference of charge layer polarity agrees qualitatively well with that of the automated Chargepol method. The vertical distance between each polarity's vertical source distribution maxima were sufficiently large by more than 1 km (Figures 2 and 3 histograms), leading to charge layers being well identified in the vertical dimension.

### 3.2 Validation Using Vertical Distribution of VHF Sources

An additional method to validate the Chargepol algorithm is the estimate of the positive charge layer altitude from the peak in the VHF source histogram (Fuchs & Rutledge, 2018; Fuchs et al., 2018; Lang & Rutledge, 2011; Lang et al., 2020). Figures 2b and 3b show an additional histogram of the vertical source density. The histogram for the normal case (Figure 2b) presents the peak at the same altitude the Chargepol method shows a peak with the most occurrences of positive charge. A comparison of these two methods shows that the Chargepol method has the advantage of inferring negative charge layer altitude, which is not possible to estimate from the LMA VHF source distribution. For the anomalous case (Figure 3b), the main low‐level peak from the anomalous charge structure storm and the secondary peak from the normal storm are depicted. The peak from the source histogram is at a slightly higher altitude, 5–5.5 km, compared to Chargepol's positive inference at 4–5 km. However, both methods generally agree and the depiction of the negative layer by Chargepol is notable.

## 4 Chargepol Method Applied to RELAMPAGO Thunderstorms

How charge is structured in Argentinian thunderstorms is generally unknown, and so we make use of the Chargepol method to examine them. During the five‐month period the RELAMPAGO LMA network was operating in Cordoba, Argentina. Different storm modes were observed and included isolated convection, multicellular storms, supercells, and mesoscale convective systems (Nesbitt et al., 2021). In order to demonstrate the capability of the algorithm to infer their charge structures, examples of distinct Cordoba cases and their evolution in time are presented. Examples of thunderstorms with different charge structures in Cordoba are shown and included normal, anomalous, a case with an enhanced lower positive charge layer, and one that demonstrated a change from one archetype to another through its lifetime. The altitudes at which either positive or negative polarities were classified most frequently, and the mean altitude of tops and bottoms of each charge layer polarity were examined every hour to show how charge layer altitude varied with time for all presented cases. The variation of a dipole's altitude with time depicts a storm's charge structure evolution and it is shown in this study in order to demonstrate a possible application a user can generate with this data set. For a lower charge layer polarity from a given dipole, the mean altitude of tops and bottoms of charge layers estimated from flashes were only calculated for charge layers in which its top was at a lower altitude than the mean upper dipole polarity altitude. Similarly, for the upper charge layer polarity from a dipole, the mean altitude of its top and bottom was obtained from charge layers with its bottom above the altitude of the mean altitude of the lower charge layer polarity. These restrictions were put in place to focus analysis on the top and bottom altitudes of the dominant positive and negative charge layers in the main dipole. To further demonstrate the algorithm's capabilities over regions of the United States that have been studied and well characterized with other charge retrieval methods (e.g., Bruning et al., 2010; MacGorman et al., 2005; Mecikalski et al., 2015; Wiens et al., 2005), an example from each of the LMA networks deployed during DC3 are shown in the Figure S1 and included a normal tripole case in Alabama, anomalous storms in Colorado, a case with a transition from anomalous to normal charge structure in Oklahoma, and a normal dipole in West Texas (negative in mid‐levels, positive in the upper levels) but with a very high altitude negative charge layer observed above the upper positive.

### 4.1 December 27, 2018 Case: Normal Charge Structure

**Figure 4:**
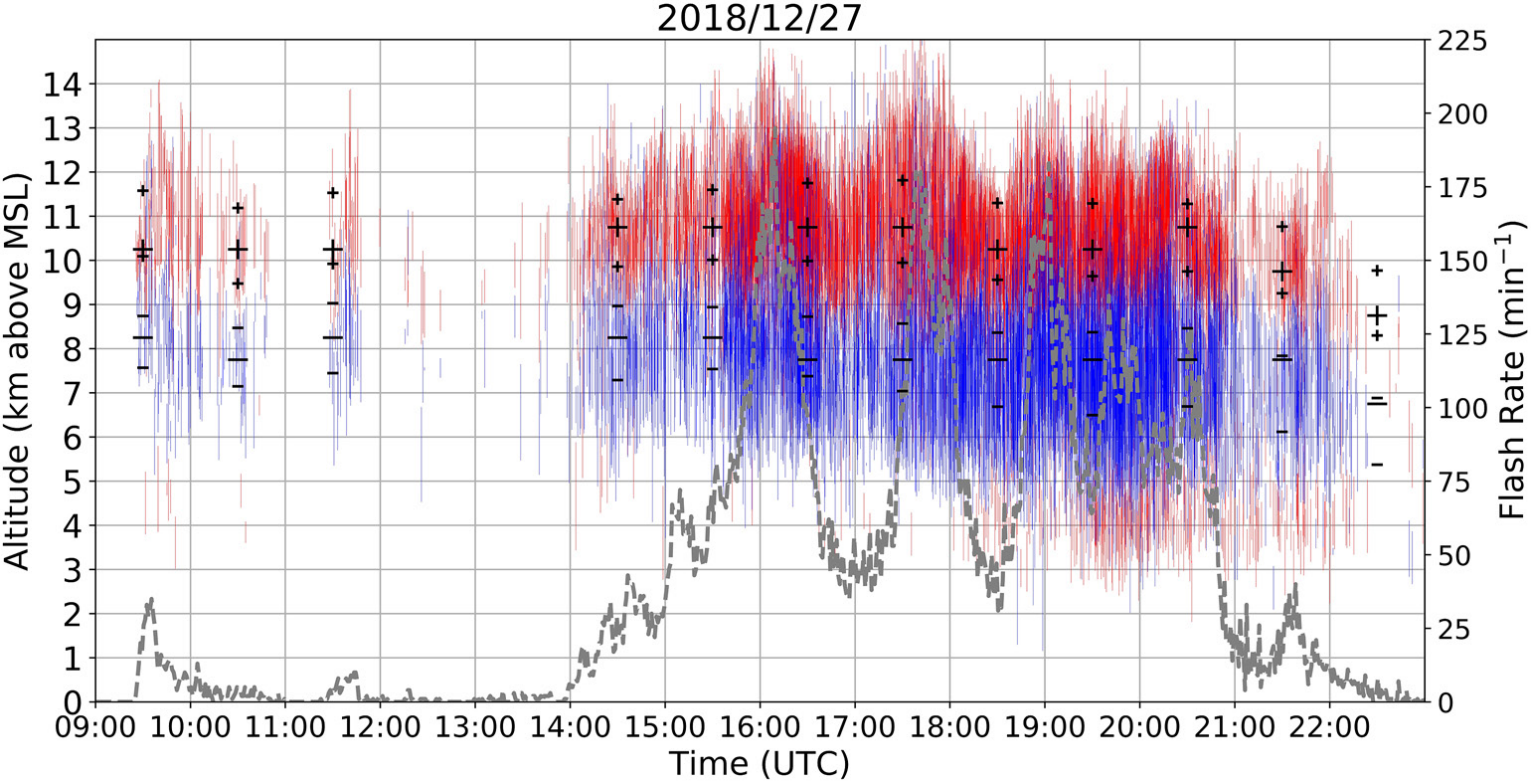
Charge layers estimated from flashes using the Chargepol automated method for all Remote sensing of Electrification, Lightning, And Mesoscale/microscale Processes with Adaptive Ground Observations (RELAMPAGO) thunderstorms on December 27, 2018 from 0900 to 2300 UTC. Each red (blue) vertical line represents a positive (negative) charge layer estimated from a flash. Large black symbols represent the altitudes in which most charge layers of a certain polarity were estimated for each hour period, as long as more than 30 layers with that polarity were present in that hour. Small black symbols represent the mean altitudes of the top and bottom of charge layers for each polarity and hour. Dashed gray line shows flash rate for the entire domain.

Figure 4 shows the estimate of charge layer polarity for all convective storms (most of them multicellular as inspected from radar data) that occurred in the RELAMPAGO LMA domain for a 14‐h period on December 27, 2018. Most thunderstorms that occurred on this day presented an upper‐level positive charge layer above 9–10 km height, and a mid‐level negative charge layer between about 5 and 9 km height. Altitude variation in charge layers is speculated to be due to different thunderstorms having varying updraft strength and cloud‐top heights. As the number of charge layers vary within a storm, where more charge layers are found where flash rates are highest (Brothers et al., 2018; DiGangi et al., 2016, for example), we inferred flash rates from periods when charge layers were estimated frequently in short periods of time. For example, more frequently estimated charge layer polarities in shorter timespans were indicative of higher storm flash rates than those less frequently estimated. For most of the period between 1500 and 2100 UTC, the total flash rates of all storms was higher than 50 flashes/minute, considering flashes with more than 10 sources and all active thunderstorms in the domain. The total flash rate of storms in the domain peaked at 195 flashes/minute at 1609 UTC. The dominance of positive over negative charge structure means that most flashes depicted by Chargepol were +ICs, with a typical initial upward motion of a negative leader and further propagation through the upper positive charge layer. This general dipole structure characterizes a typical normal charge structure, as it is common in many regions of the United States as shown in similar LMA‐based charge retrieval studies, such as in Alabama (Mecikalski et al., 2015) and Oklahoma (Bruning et al., 2007). Some flashes propagated through a lower positive charge layer below 5 km height, principally after 1920 UTC. That was caused by −IC flashes with initial negative leaders with downward motion and further propagation through the low‐level positive charge region. Hence, from 1900 to 2200 UTC, a typical tripole charge structure (Williams, 1989) was present, though the upper positive region is considerably more active than the lower positive due to more flashes contributing to the upper positive depiction.

### 4.2 March 14, 2019 and December 5, 2018 Cases: Anomalous Charge Structure

**Figure 5:**
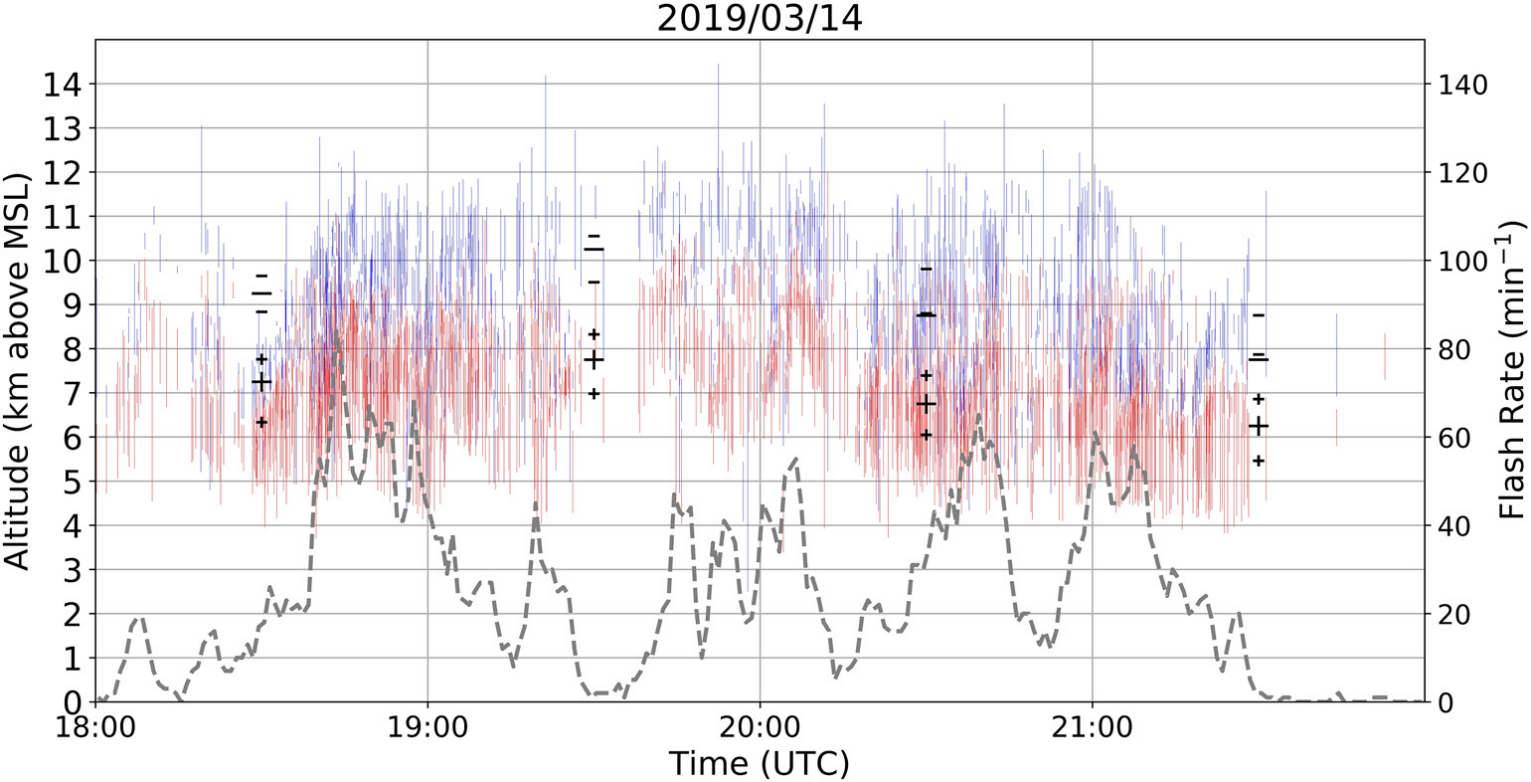
Same as in Figure 4, but for March 14, 2019 from 1800 to 2200 UTC.

**Figure 6:**
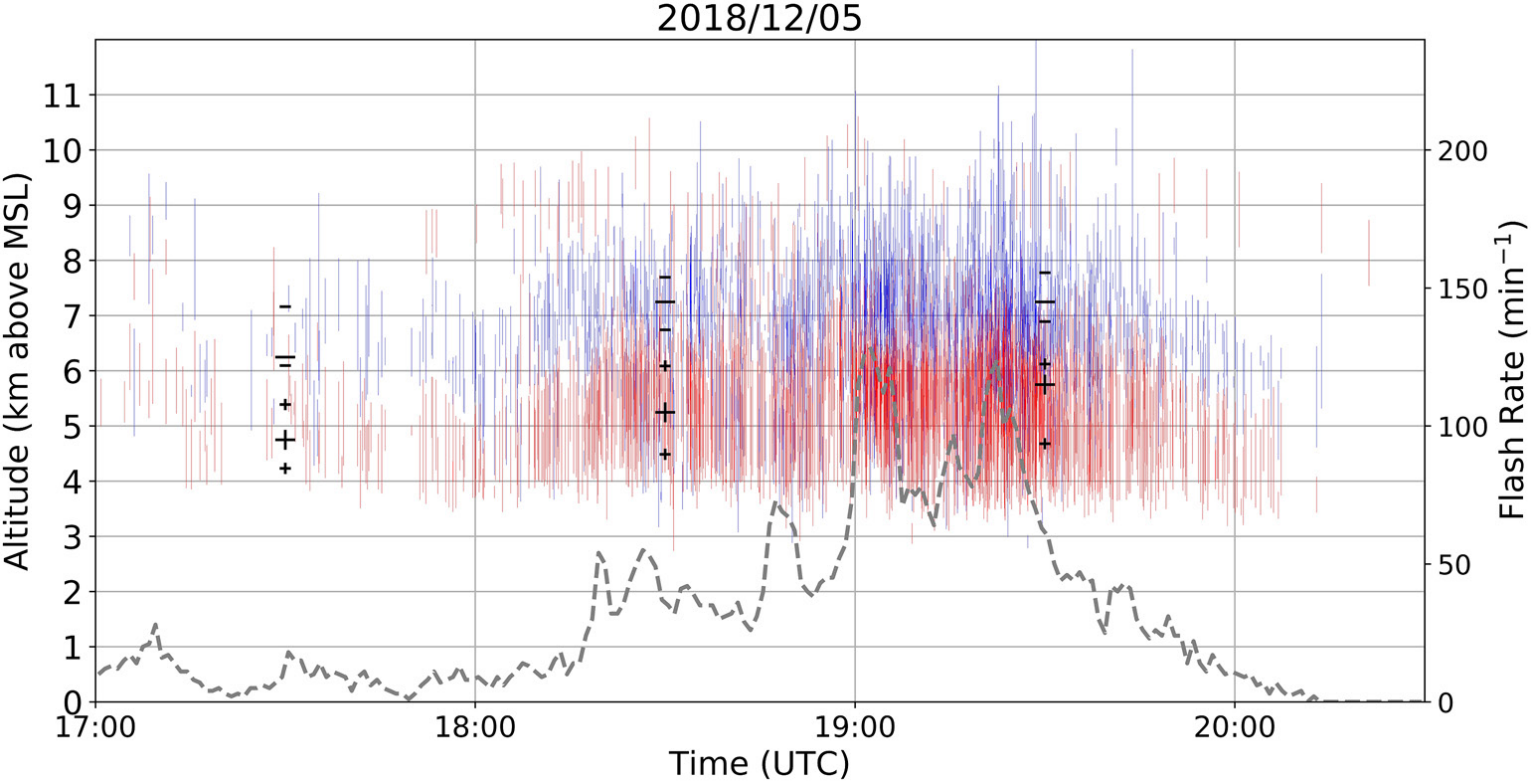
Same as in Figure 4, but for December 5, 2018 from 1700 to 2030 UTC. Dashed gray line shows flash rate for the most active storm only.

A cluster of RELAMPAGO storms on March 14, 2019, all with anomalous dipole charge structures, are shown in Figure 5. These storms had a dominant mid‐level positive charge layer and upper‐level negative charge layer, similar to some anomalous storms over Colorado (Fuchs et al., 2015). As multiple storms are shown in Figure 5, a large altitude variation is noticeable for the charge layers, which is possibly dependent on individual storm intensity. Storms with stronger updrafts are thought to initiate flashes between charge layers residing at higher altitudes (Stolzenburg et al., 1998). Most flashes in these storms presented −IC lightning, which means that negative breakdown had an initial downward propagation, hence negative charge being estimated at higher levels than positive charge. Peak flash rate was observed to be 84 flashes/minute at 1843 UTC. A similar anomalous dipole case occurred in an isolated thunderstorm on December 5, 2018 (Figure 6). This storm had a flash rate higher than 30 flashes/minute for most of the period between 1815 and 1945 UTC, with a peak flash rate of 128 flashes/minute at 1902 UTC. This anomalous case is different from the March 14, 2019 anomalous case because estimated charge layers are located at lower levels: negative charge is located in the mid‐levels, while positive charge is in the low‐levels. Also, this was a relatively shallow storm system exhibiting a radar echo top at about 10 km height (not shown), hence no upper positive charge layer had developed. Upper positive charge at about 9 km height from 1800 to 1900 UTC was from another storm in the domain (see discussion in Section 3.1).

### 4.3 March 7–8, 2019 Case: Transition From Anomalous to Normal Charge Structure

**Figure 7:**
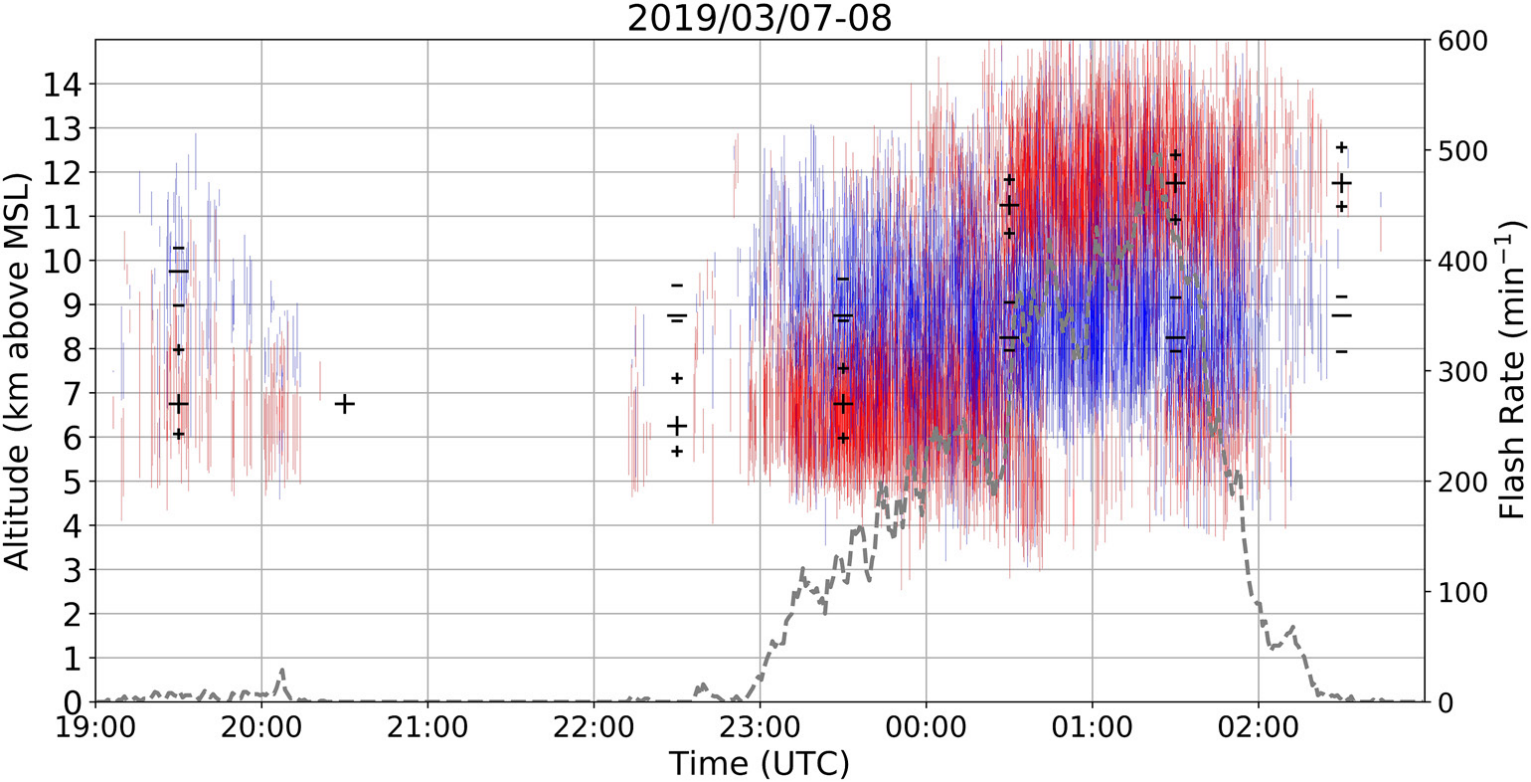
Same as in Figure 4, but for March 7, 2019 at 1900 UTC to March 8, 0300 UTC.

Thunderstorms on March 7, 2019 (Figure 7) during RELAMPAGO presented an anomalous charge structure with mid‐level positive charge and upper‐level negative charge. From 1900 to 2300 UTC, storms that occurred in the LMA domain had a low flash rate (less than 30 flashes/minute considering all thunderstorms in the domain), then few charge layers were depicted by Chargepol, but an anomalous dipole is clearly present, similar to the storm studied by Fuchs et al. (2018) over Colorado. After 2300 UTC, a MCS formed with a dominant anomalous charge structure, with its flash rate rapidly increasing to more than 100 flashes/minute in the LMA domain. On the following UTC day, high flash rates remained, reaching a peak of 496 flashes/minute at 0124 UTC in the domain, and an upper positive charge layer formed above 10 km height. This upper positive layer became visible because flashes started propagating through that layer. After 0045 UTC, fewer flashes propagated through the lower positive charge layer. Hence, this case characterizes a transition from anomalous to normal charge structure. This case demonstrates how complex charge structure evolution can be estimated by the Chargepol method, such as the presence of anomalous and normal main dipoles, tripoles, and their evolution in time.

## 5 Frequency of Anomalous Charge Structure in Central Argentina Compared to the U.S.

As the described Chargepol method allows for a relatively fast processing time for large datasets of months of LMA data, one can obtain the general charge structure evolution in time for a domain area, as shown in the previous section. Hence, in order to characterize the likelihood of normal and anomalous charge structure for the five months in which the LMA was deployed in the Cordoba, Argentina region for the first time, the Chargepol layer polarity output was summarized for a better understanding and interpretation of the general dominant charge structure.

In order to achieve a summary of the general charge structure typically occurring in Argentinian storms for long periods of time, the charge polarity information was initially subdivided into time periods of one hour to obtain the dominant dipole for every hour period. Then, the number of charge layers of a given polarity were counted for every altitude in 0.5 km bins for every hour. The altitude with the most positive charge layers estimated from flashes, and the altitude with most negative charge layers, define a single altitude bin for each layer polarity, which characterizes the dominant dipole for that hour, as long as both maximum polarities occur at different heights. A minimum threshold of 30 charge layers from each polarity occurring in one hour was applied, in order to remove the influence of thunderstorms with low flash rates contributing to the charge structure estimation. The large black symbols in Figures 4, 5, 6, 7 represent the altitude with most occurrences of a charge layer polarity for each hour, and thus the dominant dipole for an hour period.

In this study, an estimated dipole structure for a one hour period is referred to as a “sample.” Samples in which dipoles had positive located at a higher altitude than negative are referred in this study as normal charge structures (Dye et al., 1986; Williams, 1985). A normal charge structure sample could have few flashes that estimated the presence of a low‐level positive charge layer, however if more flashes contributed to the maximum height occurrence of positive being at high altitudes, it would be considered a normal charge structure sample. Figure 4 shows an example of a normal tripole charge structure (Williams, 1989) with more positive layers estimated at high altitudes, leading to a normal dipole estimation. Samples with negative charge over positive charge are considered to have a dominant anomalous charge structure. The most common type of anomalous dipole sample is the type with positive charge at the mid‐levels or mixed‐phase layer, and negative in the upper levels of a storm (Figure 5). Another structure that could lead to a negative‐over‐positive dipole is when enhanced positive charge is at low levels of a storm, while negative charge is at the mid‐levels (Figure 6, Bruning et al., 2014; Fuchs et al., 2015). In this scenario, an upper positive charge could be present, which could lead to an interpretation of a normal tripole charge structure, an uncommon characteristic during RELAMPAGO as the enhanced low‐level positive charge layer is not typically accompanied by an upper‐level positive charge layer (Figure 6). However, in this study and others (e.g., Fuchs et al., 2015), a normal tripole scenario with more flashes propagating through the lower positive charge layer than through the upper positive would imply the characterization of an anomalous dipole. Both anomalous scenarios (positive in the mid‐levels, and in the low levels) imply that most flashes were −ICs with negative leaders having an initial downward motion, rather than +ICs that would initially move upward. Hence, in this study, scenarios with a dominance of −ICs, or negative‐over‐positive dipoles, are considered anomalous.

During the five months that the LMA was operating in Cordoba, Argentina, 306 samples were observed, which means 306 h with lightning activity in which the aforementioned methodology estimated a dominant dipole structure. Among the 306 Cordoba samples, 265 consisted of normal dipole charge structure, while the other 41 were anomalous (Table 1). That means that 13.3% of samples had a dominant anomalous charge structure, which can be interpreted as an approximate frequency of occurrence of anomalous storms in Cordoba, Argentina. Table 1 shows the number of normal and anomalous samples for the Cordoba LMA deployed during RELAMPAGO, as well as for the four LMA networks deployed during DC3 in several locations across the United States (e.g., Colorado, West Texas, Oklahoma, and Alabama) for comparison, all sampled in the warm season (May and June 2012). Table 1 also shows the total number of flashes with more than 20 sources, and the fraction of flashes that were considered by the algorithm, being 16.7% when averaging all LMA networks. The comparison of Cordoba dipole samples with DC3 networks is shown to demonstrate Chargepol's usefulness and capabilities in polarity estimation, as it made estimates of charge structure climatologies similar to those observed in other studies. Even though the sample numbers vary for the different locations, consistent with Carey et al. (2003) and Fuchs and Rutledge (2018), Alabama showed the lowest percentage of anomalous storms (7.3%), and Colorado anomalous frequency was much higher than any other region (82.6%). Oklahoma and West Texas fell in between these two regions, and with similar anomalous frequencies to Cordoba (12.9% for Oklahoma and 11.1% for West Texas). From the flash centroid altitude distribution for the entire RELAMPAGO LMA data set, Lang et al. (2020), observed a peak at 10 km height and a secondary peak at 6 km height, the latter attributed to anomalous storms and stratiform lightning. For the normal and anomalous Cordoba events shown in Lang et al. (2020), Chargepol algorithm depicted the correct general dipole polarity, consistent with their analysis.

**Table 1:** Number of Normal and Anomalous Samples for Cordoba, Alabama, West Texas, Oklahoma, and Colorado

	Cordoba	Alabama	West Texas	Oklahoma	Colorado
Number of days	157	32	48	41	61
Number of flashes (>20 sources)	808,416	39,046	261,713	497,139	545,005
Number of flashes considered by Chargepol	165,767 (20.5%)	7,653 (19.5%)	65,309 (24.9%)	58,900 (11.8%)	62,556 (11.4%)
Total number of samples	306	41	99	80	98
Normal samples	265	38	88	70	17
Anomalous samples	41	3	11	10	81
% Anomalous	13.3	7.3	11.1	12.5	82.6

**Figure 8:**
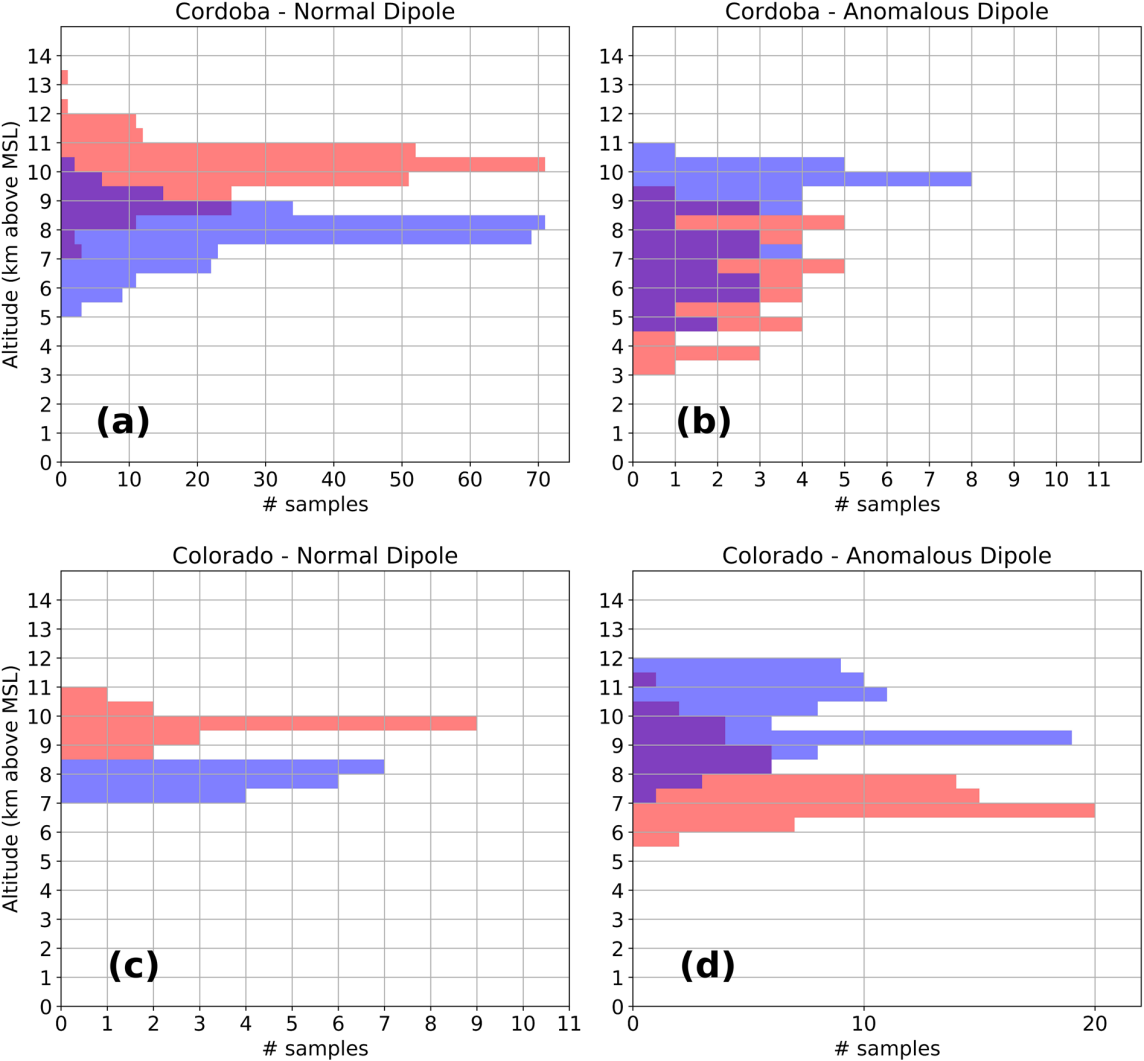
Distribution of normal and anomalous samples with altitude for Cordoba (a), (b) and Colorado (c), (d).

The distribution of normal samples with altitude demonstrated that most normal dipoles were present in the mid‐to‐upper levels; that is, with mid‐level negative and upper‐level positive charge. Figure 8a shows the distribution of normal dipoles for Cordoba, Argentina. The altitude distribution of anomalous samples (i.e., negative over positive dipoles) in Argentina shows that there were cases in which negative charge was present in the upper levels with positive in the mid‐levels, and cases of negative in the mid‐levels, with enhanced positive in the low levels (Figure 8b). In Colorado, few normal samples were observed, but their altitude distribution is similar to Cordoba (Figure 8c). The distribution of anomalous samples with altitude in Colorado showed that most dipoles had upper level negative and mid‐level positive. No apparent presence of an anomalous dipole located in the low‐mid‐levels occurred in the Colorado DC3 LMA data set. Therefore, the Cordoba December 5, 2018 case (Figure 6) demonstrates a singular thunderstorm charge structure that is, either rare or completely absent in Colorado. The normal sample distributions in height for the other three U.S. locations (not shown) were similar to Cordoba and Colorado, while the anomalous sample distribution for these 3 locations (not shown) proved inconclusive due to the low sample number.

## 6 Summary and Discussion

This paper presented charge structures for the warm season thunderstorms in Cordoba, Argentina for the first time through thunderstorm examples and long‐term statistics utilizing a new method that identifies charge layer polarity at a flash level from LMA VHF data. This method is able to estimate general charge structures such as normal and anomalous dipoles, tripoles, altitudes, and vertical depths of charge layers. Chargepol was applied for a total of 13 months of LMA data, allowing for the inference of the frequency of anomalous and normal charge structure thunderstorms in Cordoba, Argentina, and comparison to four well‐studied U.S. regions using the same methodology.

This method was developed from a meteorological standpoint, which means that the objective was to obtain the general charge structure evolution through the entire thunderstorm life cycle, or for many hours of data. In order to achieve that, there was no need to retrieve charge polarity from every flash as demonstrated in the comparison of Chargepol relative to manual charge structure analysis and the VHF source distribution peak. Instead, only flashes with less doubtful characteristics were used to provide an accurate charge polarity retrieval. Hence, when considering such long periods of time, the frequency of anomalous and normal charge structures can be estimated. Also, we found that it is sufficient to summarize the data into the main dominant dipoles for every hour in order to characterize the charge structure for a region. It is important to emphasize that, once charge layers are retrieved from individual lightning flashes, one can organize this same data set in any other manner depending on the user's purpose. Examples include considering the algorithm output as a database to be organized into shorter or longer time periods, obtaining the density of charge layers polarity over the time‐altitude domain, calculating statistics for comparison with observations from other instrumentation such as radar, etc.

The complexities of a three‐dimensional charge structure that may be present at sub‐storm scale, with charge layers extending through different altitudes depending on distance to an updraft core, are not being fully accounted for in this study. For a flash analysis, we consider the charge distribution over the vertical dimension only, which proved to be sufficient for this study's objectives. For a given flash, the Chargepol method can estimate no more than two charge layers with opposite polarities. However, when observing charge layers output for numerous flashes, it is possible to infer the presence of dipoles, their altitude and time evolution, the presence of tripoles and even multiple charge layers if flashes propagate through it. Only charge layers that had flashes moving through them can be inferred. In the case of a positive charge layer without a lightning flash moving through it, the charge layer cannot be visualized as a product of the algorithm, which is a fundamental limitation of all LMA‐based charge retrieval methods (Rust et al., 2005). The fact that Chargepol neglects small flashes for charge layer estimation, as it discards flashes with less than 20 sources, makes it hard to locate small pockets of charge within thunderstorms. Even if these charge regions were located, it could be hard to visualize and interpret their evolution over minutes. Also, differentiating charge structure of small flashes from noise would be challenging, an issue to be addressed in a future study. In order for small flashes to be included in the analysis for identification of finer charge structures, threshold of parameters have to be relaxed prior to running Chargepol. However, estimating the general dipole and tripole charge structures is feasible with the conditions used in this study, satisfying our purpose.

The Chargepol method proved capable for analyzing large LMA datasets in a reasonable processing time of minutes, allowing for efficient interpretation of charge structures over Cordoba, Argentina during the recent RELAMPAGO field campaign and a consistent comparison of these novel results with thunderstorms from different regions of the United States whose charge structures have been sampled with LMA and are well understood. A high frequency of anomalous storms were found for Colorado, consistent with other studies (Fuchs et al., 2018). Examples of Cordoba anomalous thunderstorms with altitude distributions of positive charge layers that are uncommon in Colorado were presented. Interestingly, Cordoba showed slightly higher anomalous charge structure frequency compared to Oklahoma and West Texas, while Alabama presented the lowest anomalous frequency among all studied regions consistent with prior work (Fuchs et al., 2018). Reasonings for these results were not explored in this study. The meteorological, environmental, kinematic and microphysical conditions in Central Argentina are speculated to be important contributors to the observed charge structures documented herein during RELAMPAGO, and they will be explored in future studies and compared to past work from other regions throughout the world. The charge polarity outputs presented in this study have the potential to be useful for numerous applications in lightning research, and Chargepol has been made available as an open‐source algorithm with options to choose parameter thresholds.

## Supporting information

Supporting Information S1

## Data Availability

RELAMPAGO LMA data are available on https://doi.org/10.5067/RELAMPAGO/LMA/DATA101. NSF DC3 LMA data are available on https://data.eol.ucar.edu/master_lists/generated/dc3/. Chargepol algorithm is available at https://github.com/brmedin/chargepol.
