# Supplementary material for: Characterizing Charge Structure in Central Argentina Thunderstorms During RELAMPAGO Utilizing a New Charge Layer Polarity Identification Method
Source: Earth Space Sci. 2021 Aug 18;8(8):e2021EA001803. doi: 10.1029/2021EA001803 (PMC8459255; doi:10.1029/2021EA001803)
Supplement: Supplementary file 1 — Supporting Information S1 [file ESS2-8-e2021EA001803-s001.pdf]

AGU PUBLICATIONS

Earth and Space Science

Supporting Information for

Characterizing Charge Structure in Central Argentina Thunderstorms During RELAMPAGO Utilizing a New Charge Layer Polarity Identification Method

Bruno L. Medina[1], Lawrence D. Carey[1], Timothy J. Lang[2], Phillip M. Bitzer[1], Wiebke Deierling[3], and Yanan Zhu[4]

[1]Department of Atmospheric and Earth Science, The University of Alabama in Huntsville, Huntsville, AL, USA.

[2]NASA Marshall Space Flight Center, Huntsville, AL, USA.

[3]University of Colorado Boulder, Boulder, CO, USA.

[4]Earth System Science Center, The University of Alabama in Huntsville, Huntsville, AL, USA.

Corresponding author: Bruno Medina (blm0032@uah.edu)

Contents of this file

Figures S1 to S4

Introduction

Supporting information shows the charge structure evolution for a case from each of the lightning mapping arrays (LMAs) deployed during the NSF Deep Convective Clouds and Chemistry (DC3) field campaign, as inferred by the Chargepol algorithm. Cases include: a normal tripole charge structure in Alabama, an anomalous dipole charge structure in Colorado, transition from anomalous to normal charge structure in Oklahoma, and a normal dipole with an additional negative charge at high levels in West Texas.

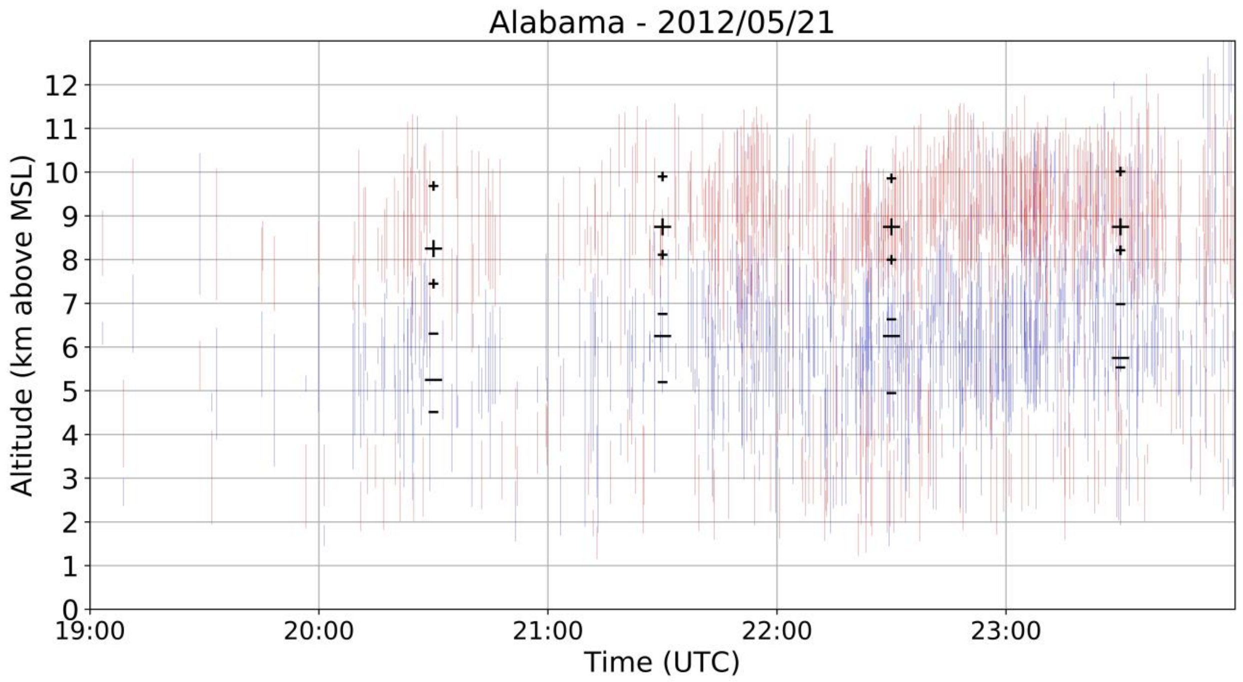

Figure S1. Charge layers estimated from flashes using the Chargepol automated method for all thunderstorms in Alabama on 21 May 2012 from 1900 to 0000 UTC, demonstrating a normal dipole/tripole case. Each red (blue) vertical line represents a positive (negative) charge layer estimated from a flash. Large black symbols represent the altitudes in which most charge layers of a certain polarity were estimated for each hour period, as long as more than 30 layers with that polarity were present in that hour. Small black symbols represent the mean altitudes of the top and bottom of charge layers for each polarity and hour.

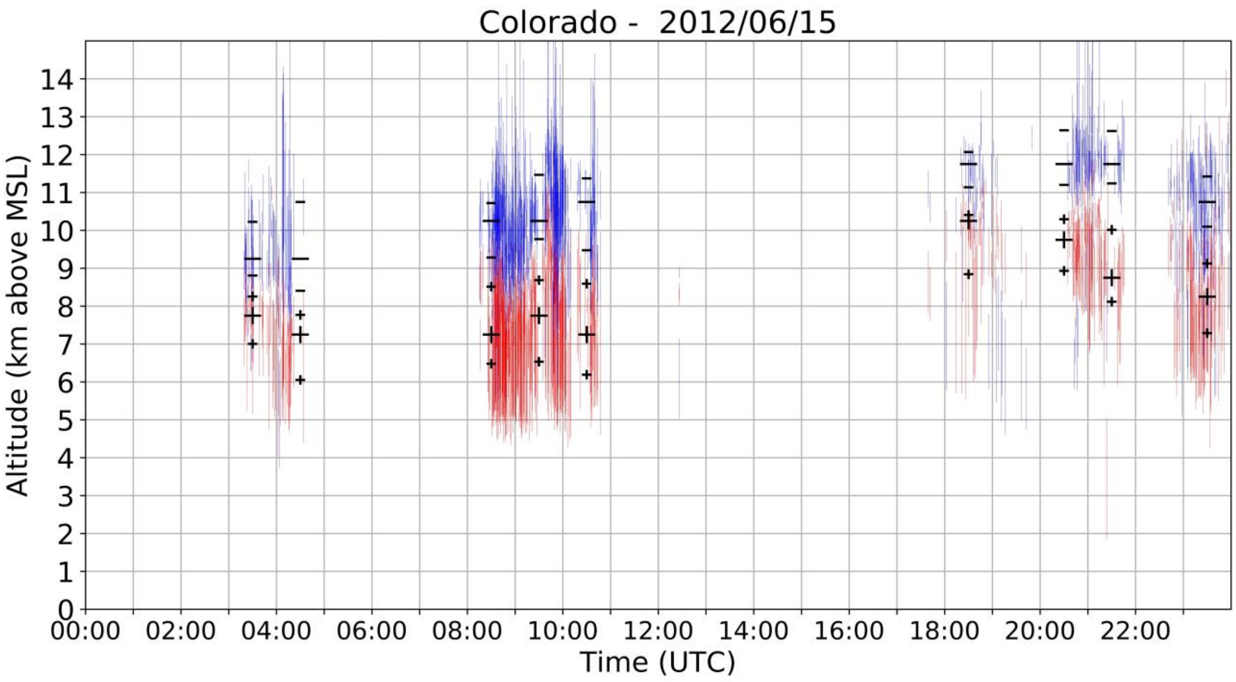

Figure S2. Same as in Figure S1, but for Colorado in 15 June 2012, demonstrating anomalous charge structure storms.

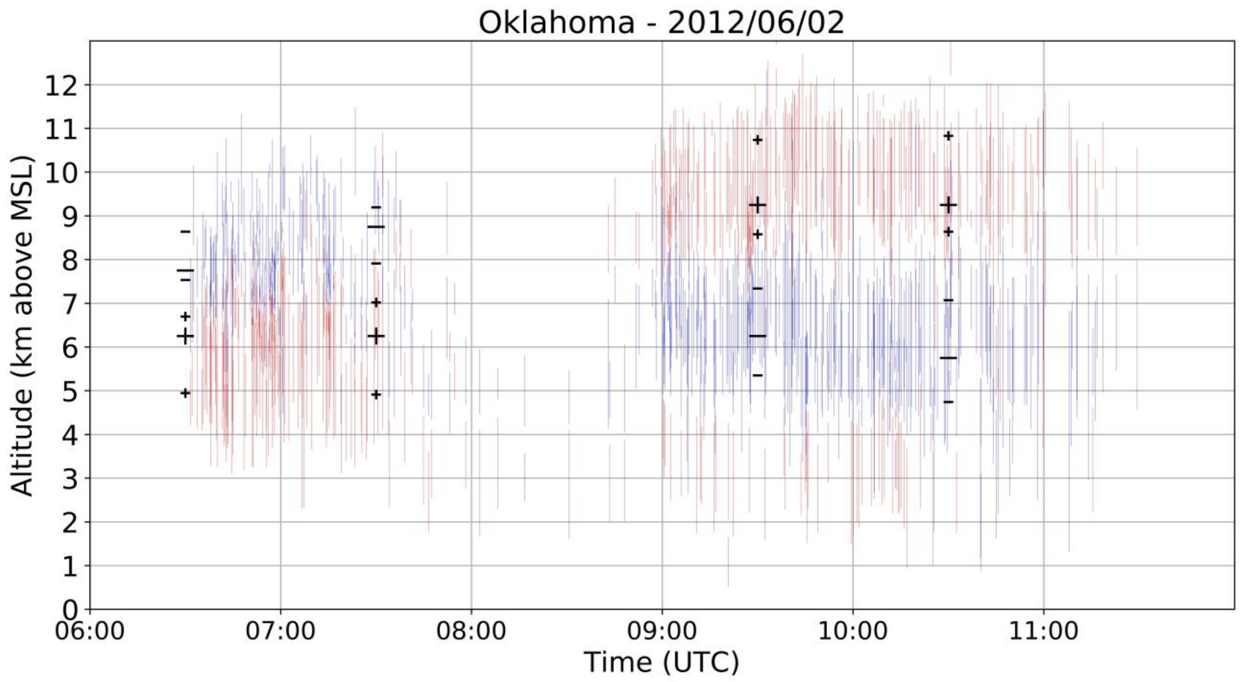

Figure S3. Same as in Figure S1, but for Oklahoma in 2 June 2012 from 0600 to 1200 UTC, showing a transition from anomalous to normal charge structure case.

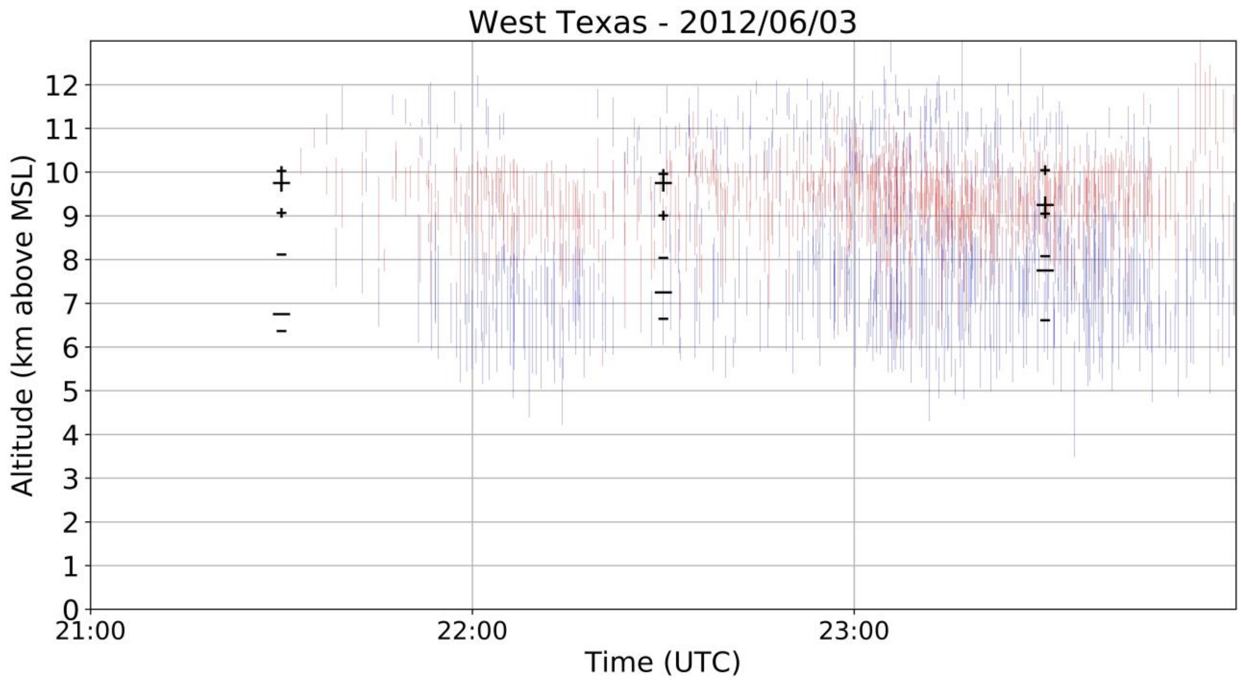

Figure S4. Same as in Figure S1, but for West Texas in 3 June 2012 from 2100 to 0000 UTC, showing a normal dipole charge structure with an additional negative charge layer detected above the main positive charge.
